# Micro Data Analysis of Medical and Long-Term Care Utilization Among the Elderly in Japan

**DOI:** 10.3390/ijerph7083022

**Published:** 2010-07-30

**Authors:** Hideki Hashimoto, Hiromasa Horiguchi, Shinya Matsuda

**Affiliations:** 1 Department of Health Economics and Epidemiology Research, The University of Tokyo School of Public Health, 7-3-1 Hongo, Bunkyo, Tokyo 113-0033, Japan; 2 Department of Health Management and Policy, The University of Tokyo Graduate School of Medicine, 7-3-1 Hongo, Bunkyo, Tokyo 113-0033, Japan; E-Mail: hiromasa-tky@umin.ac.jp; 3 Department of Preventive Medicine and Community Health, University of Occupational and Environmental Health, 1-1 Iseigaoka, Yahata-nishi-ku, Kitakyushu, Fukuoka 807-8555, Japan; E-Mail: smatsuda@med.uoeh-u.ac.jp

**Keywords:** end-of-life medical cost, long-term care, aging, survivorship, Japan

## Abstract

Japan is currently experiencing the most rapid population aging among all OECD countries. Increasing expenditures on medical care in Japan have been attributed to the aging of the population. Authors in the recent debate on end-of-life care and long-term care (LTC) cost in the United States and Europe have attributed time to death and non-medical care cost for the aged as a source of rising expenditures. In this study, we analyzed a large sample of local public insurance claim data to investigate medical and LTC expenditures in Japan. We examined the impact of aging, time to death, survivorship, and use of LTC on medical care expenditure for people aged 65 and above. On the basis of these findings, we conclude that age is a contributing factor to the rising expenditures on LTC, and that the contribution of aging to rising medical care expenditures should be distinguished according to survivorship.

## 1. Introduction

### 1.1. Population Aging and End-of-Life Medical Care Costs

Rising expenditures on formal services associated with medical and long-term care (LTC) in aging societies has led researchers and policy makers to examine the use of medical services prior to death. An early study of Medicare data in the United States by Lubitz and Prihoda [1] estimated that end-of-life medical expenditures accounted for 28% of the total annual medical expenditures in the country. According to their results, the majority of this amount was spent within one month before death. Other studies using updated Medicare data [2,3] have consistently reported that, in spite of the recent introduction of alternative services such as hospice and homecare, the share of end-of-life medical expenditures has remained almost constant over time. Barnato, *et al.* [4] attributed the increase in medical expenditures to an increase in the intensity of treatment under inpatient care offsetting the decrease in in-hospital deaths.

In Japan, rising expenditures on medical and LTC services have evoked a serious policy debate, wherein the aging of the population is widely considered to be the major driving force behind these increasing costs. Of all OECD countries, Japan has experienced the most rapid rate of population aging in the recent period. In 2008, the proportion of the population aged 65 and over had already reached 22% of the total population. As such, it is imperative that policy makers in Japan understand precisely how, and to what extent, population aging influences expenditures on medical care and LTC.

Ogura, *et al.* [5] produced the first estimate of end-of-life medical expenditures in Japan, using claim bill data collected from local public health insurers in the late 1980s. They estimated that the share of end-of-life medical expenditure for people aged 70 and over was 19.2% of annual medical expenditures. Fukawa [6] conducted a similar analysis using claim data collected from 12 prefectures in the early 1990s for beneficiaries aged 70 and over. It was found that the proportion of end-of-life medical care (inpatient and outpatient services) was 12% of the total annual expenditure, and that the majority of this amount was spent within the three months immediately prior to death. This increase in expenditures before death was attributed to two factors: an increase in the probability of admission and an increase in cost per case. A study by Konno [7] examined Employee Health Insurance claim bill data from the late 1990s, and estimated that the proportion of end-of-life expenditure was 22.4% of the total expenditure for those aged 65 and over. In addition, this study also reported that the medical expenditures of decedents compared to the medical expenditure of survivors was about 5.3 times greater for those aged 70 and over. The Institute of Health Economics and Policy [8] estimated that the average medical expenditure one month prior to death was approximately 1,120,000 Japanese Yen (JPY; equivalent to $11,200 US at an exchange rate of 1 USD = 100 JPY). The Bureau of Insurance in the Ministry of Health, Welfare, and Labour multiplied this number by the estimated number of hospital deaths in 2002 (approximately 0.8 million), arriving at an estimate of annual “end-of-life expenditure in inpatient service” for 2002 of 900 billion JPY, or 9 billion US dollars.

### 1.2. Review of the “Red herring” Debate

It has been proposed by several authors that the figure announced by the Ministry is an overestimate, because end-of-life medical expenditure declines with age [3,6,7,9]. The decline in end-of-life medical care expenditures among the older old is thought to be caused by a decrease in the probability of service use [10], or a decrease in the intensity of care [11,12]. Thus, projections of the growth in medical expenditures produced by simply multiplying the current age-specific medical expenditure by an estimate of future population are likely to be overestimated compared to estimates based on more sophisticated models that take end-of-life expenditure into consideration [13,14].

In the so-called “red herring” debate [15], it has been argued that time-to-death (TTD), rather than population aging *per se*, is the cause of rising medical expenditure. Subsequent studies using more sophisticated econometric techniques [10,16], confirmed that TTD was a significant factor, although, it was argued that aging is also an important contributing factor to rising medical expenditures.

The studies discussed so far focused only on medical services, and ignored the cost of LTC. As such, they do not provide a complete picture of the total healthcare costs of the elderly before death. Hoover, *et al.* [17] used the 1992–1996 Medicare Current Beneficiary Survey data and found that although medical expenditure on end-of-life care was lower in the elderly, non-Medicare expenditure was higher for older beneficiaries. Further, they found that end-of-life medical expenditures increased sharply during the month of death whereas non-Medicare expenditure was stable over the year. They concluded that custodial and chronic care, which is covered by non-Medicare expenditures, should also be seriously considered to improve resource allocation for end-of-life care of the elderly. Using the claim data of dual beneficiaries for Medicare and Medicaid in 10 states, Liu, *et al.* [18] confirmed this trend in medical care and LTC expenditures before death.

Polder, *et al.* [14] used a large representative sample of public insurance data from The Netherlands to estimate the total healthcare costs, including the cost of medical care and LTC. They confirmed that the cost of medical care decreased with age for decedents, whereas the cost of LTC increased with age. In addition, they found that elderly survivors spent less on medical care compared to decedents, but their medical cost did not vary across age groups. Furthermore, since LTC among elderly survivors increased with age, the healthcare cost of survivors aged 80 and over was about half of the total healthcare cost of decedents. Thus, they concluded that aging *per se* could not be ignored as a contributing cause of rising healthcare expenditures.

On the basis of Swiss claim data, Werblow, *et al.* [19] proposed that the impact of TTD and age on end-of-life healthcare cost differs according to survivorship and use of LTC. Although the annual medical expenditure for decedents who did not use LTC was extremely high, this expenditure decreased with age. In contrast, for survivors without LTC this expenditure increased with age until the age of 85, but decreased thereafter. For decedents and survivors who used LTC, expenditure was lower as compared to the expenditure of long-term non-users, but increased monotonously with age. However, the Swiss claim data used in this previous study was limited to the medical component of LTC, and did not include the cost of custodial care at home or the accommodation cost incurred at nursing homes.

### 1.3. Purpose of the Study

A review of the current literature raises a number of unanswered questions. For example, the impact of further aging of the population on medical and LTC expenditure is currently unclear. Similarly, it is unclear how the use of formal LTC, including homecare and institutional care, affects medical expenditures on the elderly. Since East Asian countries are currently facing the most rapid increase in population aging in the World, any study seeking to answer these questions for Japan will also be relevant for other countries in this region.

Data from Japan are unique and appropriate for conducting such an analysis for several reasons. First, Japanese public medical insurance provides a comprehensive coverage for outpatient and inpatient medical services that cover the cost of physicians, hospitals, drugs, laboratory examinations, dental care, and surgical equipment. Medical insurance does not cover services that are regarded as less essential, e.g., health check-ups, eyeglasses, and over-the-counter pharmaceuticals. Japanese LTC insurance provides coverage for formal services to provide custodial care at home by professional helpers, respite services, outpatient rehabilitation service, visiting nurse services, and institutional care at nursing homes. Care at nursing homes does not include medical care, except for limited items, and mainly comprises custodial care. At the time of data collection, meal and accommodation expenses at nursing homes were also covered under institutional care. Unlike the system in Germany, Japanese LTC insurance does not cover cash benefits to informal caregivers.

Although private insurance providers exist in Japan, the current law permits them to cover only a limited proportion of out-of-pocket payments. With the national fee schedule and the fee-for-service basis of reimbursement from medical and LTC insurance, it would thus be expected that claim data for public insurance would provide a precise and comprehensive picture of the expenditure incurred by providing formal medical and LTC services to the elderly. Importantly, this would be the first detailed analysis of total healthcare cost for the elderly in a non-European setting based on such a large database.

In the current study, we estimated the total health cost of end-of-life care for the elderly in Japan, and compared the expenditure pattern over the course of TTD across service types. In the following analysis, we divided medical service into outpatient and inpatient services, and divided LTC into non-institutionalized care (or homecare) including respite services, and institutional care at nursing homes. In addition, we also compared the annual expenditures on decedents and elderly survivors, and studied how the survivorship, use of LTC, and age affected expenditure for each type of service. On the basis of these findings, we propose that age is a contributing factor in the rising expenditures on medical care and LTC, and that LTC is not a cheaper substitute for medical care.

## 2. Methods

### 2.1. Data Sources

Claim data was obtained from a public authority in a prefecture in the Kyushu district in the southern part of Japan. The data covered claim data for medical care and LTC by National Health Insurance for beneficiaries aged 65 and over in the prefecture from 2000 to 2004. In Japan, National Health Insurance is a public insurance offered by municipalities, and covers self-employed and retired persons. Employees of large companies are covered by the Employee Health Insurance, and are not included in this dataset. Medical insurance covers hospital fees, physician fees, and prescription fees. In this study, we excluded fees associated with dental services from the analysis.

The data included a unique identifier, the age and sex of each beneficiary, the type of service used, the month during which the service was used, monthly expenditures on the use of the service, and exit information (death or move-out). Using the unique identifier and month, we prepared panel data that served as the master database. From this master database, we prepared two sub-datasets for analysis. The first dataset was used for the analysis of decedents. For this purpose, we first identified people who had died in 2001, 2002, or 2003, then prepared their claim data over the 12 months prior to the month of the death. This dataset included 50,857 decedents.

The second dataset was used for conducting a comparative analysis between decedents and survivors. We identified people who were alive in March 2004, and collected their annual expenditure by type of service used during April 2002 to March 2003. Thus, as reflected in the claim data, these elderly people had survived for at least 12 months after they had used the service. This dataset included 364,484 survivors.

For brevity, the sample population was categorized into three age groups: 65–74, 75–84, and 85 plus. Medical care was subcategorized into outpatient and inpatient care. Prescription and drug fees were included either in outpatient or inpatient fees, depending on where the prescription was issued. In Japan, until the 1980s, drugs were sold by clinics or hospitals through prescription. The government then introduced a policy to promote divisionalized drug sale at pharmacies and to prevent over-prescription by clinics. However, in rural areas with few pharmacies, some clinics continue to sell drugs. Therefore, in this study, we combined prescription and drug fees with medical care fees. LTC was subcategorized into home care (including respite care) and institutional care at nursing homes as previously described.

### 2.2. Analysis

We followed the two-part model by estimating: (1) the probability of service use, and (2) the amount of expenditure that was conditional on the use of the service. We adopted a strategy similar to that of Polder, *et al.* [14], and relied on descriptive analysis instead of econometric modeling techniques. We chose to do so because the available dataset contained limited information on the beneficiaries, such as age and sex, so the explanatory power of any regression model would be expected to be weak. In addition, simple descriptive statistics on service use are easier to comprehend.

For conducting the analysis of the decedents, we calculated the probability of service use and the amount of monthly expenditure conditional on service use by age category and month-to-death. The results were plotted on a graph to show trends in probability of use and monthly expenditure over the 12 months prior to death. The monthly probability of service use for decedents (P. _jt_) was estimated for each age category using the formula below:

(1) $$P_{\cdot jt}=\frac{1}{\text{Nd}}\sum_{\text{i}}{\text{I}}_{\text{ijt}}$$

where:

I_ijt_ = 0 if the i_th_ patient did not use the j_th_ service in the t months before death
I_ijt_ = 1 if the i_th_ patient used the j_th_ service in t months before death
  t = 0 ∼ 11 months to death
  j = 1 ∼ 4 (1 = outpatient, 2 = inpatient, 3 = homecare and 4 = institutional care)
  Nd = total number of decedents in the age category

Monthly expenditure conditional on service use for decedents (C.jt) was estimated as:

(2) $$C_{\cdot jt}=\sum_{\text{i}}{\text{C}}_{\text{ijt}}/\sum_{\text{i}}{\text{I}}_{\text{ijt}}$$

where C_ijt_ = service use (in JPN) by the i_th_ patient for the j_th_ service in the t months before death.

For a comparative analysis of the decedents and the survivors, we calculated the annual expenditure for each type of service. When calculating the probability of use, no service use in a year was set to 0. The estimated probability of use and the amount of service use conditional on use were plotted for different age categories by survivor status. A similar analysis was conducted for the use of outpatient services and inpatient services. This was further stratified by survivorship and use of LTC (as in Werblow, *et al.* [19]). In a formula, the annual probability of service use (AP._jk_) for decedents and survivors was estimated for each age category with the formula:

(3) $${AP}_{.jk}=\frac{1}{{\text{N}}_{\text{k}}}\sum_{\text{i}}{\text{AI}}_{\text{ijk}}$$

where:

AI_ijk_ =0 if the i _th_ patient in k _th_ status did not use the j _th_ service during the targeted 12 months
AI_ijk_ = 1 if the i _th_ patient in k _th_ status used the j _th_ service during the targeted 12 months
  j = 1∼4 (1 = outpatient, 2 = inpatient, 3 = homecare and 4 = institutional care)
  k = 1∼2 (1 = decedent 2 = survivor)
N_k_ = total number of decedents (k = 1), or of survivors (k = 2) in the age category

Annual expenditure conditional on service use by decedents and survivors (AC._jk_) was then estimated for each age category by:

(4) $${AC}_{\cdot jk}=\sum_{\text{i}}{AC}_{\text{ijk}}/\sum_{\text{i}}{AI}_{\text{ijk}}$$

where AC_ijk_ = service use (in JPN) by the i _th_ patient with the k_th_ status for the j_th_ service in the targeted 12 months

Finally, the average annual expenditure of service use (E(AC._jk_)) for the j _th_ service use among k_th_ status patients was obtained by:

(5) $$\text{E}({\text{AC}}_{\cdot \text{jk}})={\text{AP}}_{\cdot \text{jk}}\times {\text{AC}}_{\cdot \text{jk}}$$

The estimated value was analyzed by age and survivorship categories. Since the decedents were drawn from a three-year period, we first divided the total number of decedents by 3. Next, we multiplied this number by the average annual expenditure of the decedents to obtain the annual total health cost for decedents. This number was compared with the corresponding annual cost obtained for survivors.

## 3. Results

Table 1 shows the basic characteristics of the elderly decedent and survivor beneficiaries. The data showed that decedents were older than the survivors. The majority of decedents (nearly 70%) died in hospitals. This number was lower than the estimated proportion of 81% for the whole Japanese population reported by Ikegami [20] using 2002 vital statistics. Because our sample was limited to elderly cases, the proportion of institutional deaths against hospitals deaths would be expected to be higher than Ikegami’s estimate. We had information on the cause of death for a sub-sample of the decedents. The data from this sub-sample showed that 22.1% of deaths were caused by neoplasm, 13.9% by stroke, and 12.1% by heart disease.

**Figure 1.**
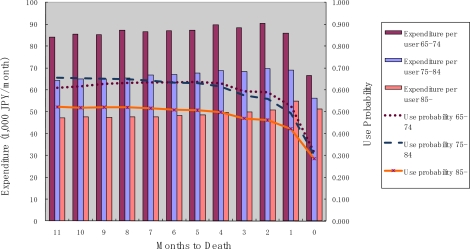
Outpatient service use and months to death.

**Figure 2.**
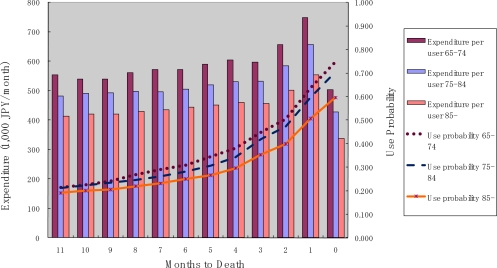
Inpatient service use and month to death.

**Figure 3.**
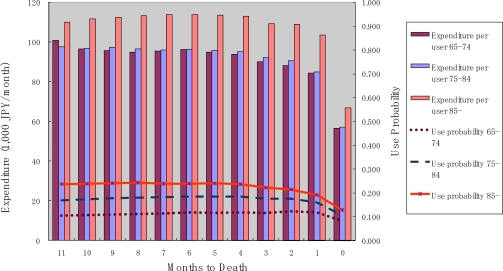
Homecare use and months to death.

**Figure 4.**
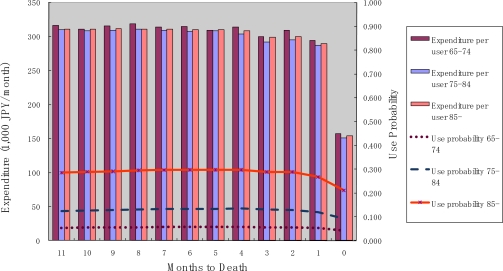
Institutional care service use and months to death.

Figure 1 shows the probability of outpatient service use, and monthly expenditures among those who used the service, for each month until death. The probability of use was constant over time, but decreased during the last two months. Monthly expenditures conditional on service use were almost constant until the month of death. Older decedents exhibited a lower probability of use and lower monthly expenditure over the period until death. It should be noted, however, that the expenditure during the month of death does not necessarily cover the full month (*i.e.*, if the death occurred in the middle of the month). This is also true for the data shown in the following Figures 2, 3, and 4.

Figure 2 shows the probability of use and monthly expenditure conditional on service use for inpatient services over the months to death. It can be seen that the probability of use and monthly expenditure increased gradually over the time period, but drastically rose in the two months prior to death, particularly in the last month before death. This trend is similar to that reported in previous studies. Again, older decedents had a lower probability of use as well as lower monthly expenditure over TTD.

Figure 3 shows the probability of use and monthly expenditure conditional on service use for homecare services over the months to death. Both the probability of use and monthly expenditure conditional on service use were constant over the months until death. In contrast to the case for medical services, the probability of use and the amount of monthly expenditure conditional on service use were highest for the oldest age category.

Figure 4 shows the probability of use and monthly expenditure conditional on service use for institutional services over the months to death. The probability of use and monthly expenditure conditional on service use were both constant over months until death. This trend is similar to the trend observed for homecare services. However, monthly expenditure conditional on service use did not differ across age categories. This is an important finding with respect to institutional care. Since institutional costs comprise custodial care and accommodation, they should be dependent only on the level of functioning and not on age.

**Figure 5.**
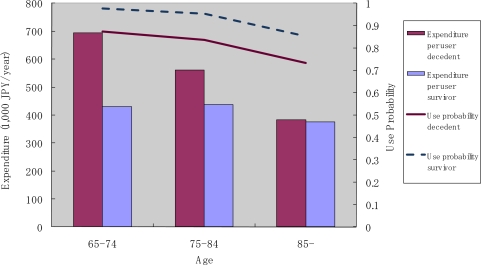
Outpatient service use by survivorship and age.

Figure 5 shows the annual use of outpatient care services by decedents and survivors. We found that the probability of use decreased with age, and that it was consistently higher for survivors than for decedents. The amount of monthly expenditure conditional on service use decreased with age for decedents, though, for survivors, a decline with age was not apparent.

**Figure 6.**
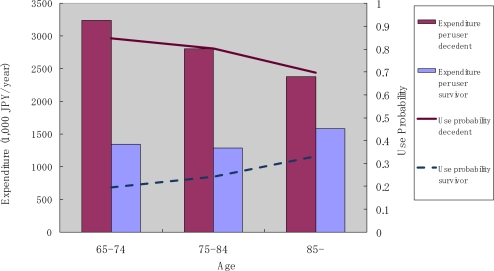
Inpatient service use by survivorship and age.

Figure 6 shows the annual use of inpatient care services by decedents and survivors. Two observations in these results are particularly notable. First, the probability of use and the monthly expenditure conditional on service use were both higher for decedents compared to survivors. Second, both the probability of use and monthly expenditure conditional on service use decreased in the older age categories for decedents, but increased with age in the case of elderly survivors.

**Figure 7.**
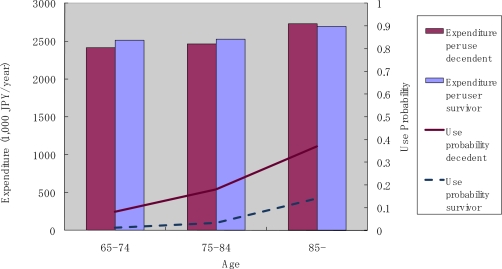
Institutional care use by survivorship and age.

Figure 7 shows data on the annual use of institutional care. Two observations are particularly notable here. First, the probability of use and monthly expenditure conditional on service use increased with age. Second, decedents had a higher probability of service use compared with survivors, but the monthly expenditure conditional on service use was similar between decedents and survivors. The trend in the use of homecare services was similar to the trend for institutional care.

**Figure 8.**
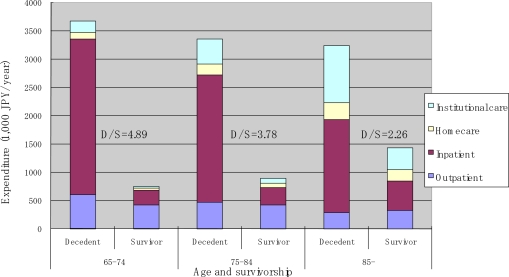
Average annual expenditure by service type, age and survivorship.

Figure 8 summarizes average annual expenditure by age, service type, and survivorship. Table 2 presents the estimated average annual expenditure by age, sex, service type, and survivorship. For decedents, the average expenditure on medical care (outpatient and inpatient services) decreased sharply with age. However, this decline was offset by an increase in expenditure on LTC. As a result, there was only a moderate decrease in total healthcare cost with age. In contrast, the average total healthcare cost monotonously increased with age for survivors, primarily because of an increase in the cost of inpatient care and LTC.

**Figure 9.**
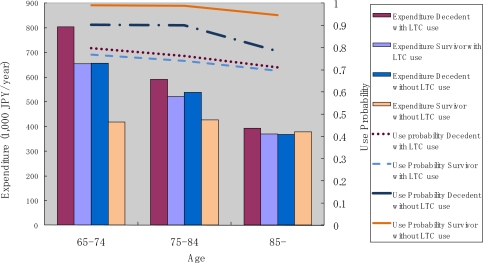
Outpatient service use by age, survivorship and LTC use.

**Figure 10.**
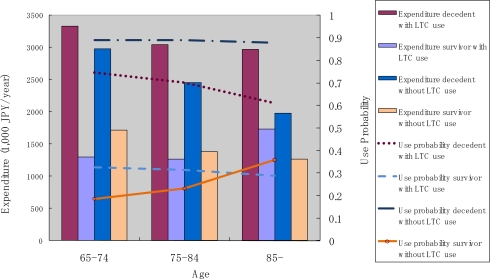
Inpatient service use by age, survivorship and LTC use.

**Figure 11.**
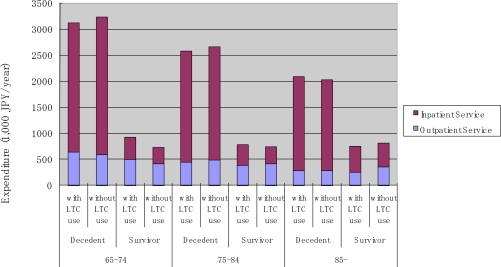
Average medical service use by age, survivorship and LTC use.

To compare our findings with those reported in The Netherlands [14] and the Switzerland [19], we conducted a similar analysis on the use pattern of outpatient and inpatient services stratified by the use of LTC. As mentioned earlier, the comparison should be interpreted with some caution because LTC in the Swiss study only included the medical component of LTC, while custodial care was included in the present report and the study in the Netherlands. In the Japanese context, people solely using medical care during the targeted 12 months may have experienced an acute onset of illness and health shock, while those who used both medical and LTC were more likely to suffer from a functional disability and gradual decline in their health status. These results are presented in Figures 9, 10, and 11. We found that the probability of use of outpatient service was the highest among survivors without LTC use, followed by decedents without LTC use, then by survivors and decedents with LTC use. Expenditure conditional on outpatient service use was higher for LTC users, and decreased over age (see Figure 9).

As was the case for inpatient services (see Figure 10), the probability of use was more dependent on survivorship than LTC use. In fact, LTC use status did not seem to affect the probability of use among survivors. In the case of decedents, LTC users were less likely to use inpatient services as compared to LTC non-users. The pattern of annual expenditure conditional on service use for inpatient services appears to be more complicated. Decedents incurred a higher annual expenditure than survivors. Decedents with LTC use incurred the largest expenditure, and the amount varied only slightly across the different age categories. Decedents without LTC incurred the second largest annual expenditure conditional on service use, and this amount decreased sharply with age. For survivors, expenditure increased slightly with age among LTC users, and decreased slightly with age among LTC non-users.

However, as shown in Figure 11, annual average expenditure did not vary across age categories in the case of survivors, but decreased sharply across age categories in case of decedents, regardless of LTC use.

Finally, we estimated the proportion of total health cost attributed to decedents, divided by age group. We found that the share was 0.100 for the 65–74 years age group, 0.124 for the 75–84 years age group, and 0.217 for the 85 plus age group. Medical care and LTC each had almost the same share in the total health cost of decedents across age groups.

## 4. Discussion and Conclusions

Surprisingly, despite differences in healthcare systems and insurance coverage between The Netherlands and Japan, our findings are relatively similar to those of Polder, *et al.* [14]. Elderly survivors were found to require less spending on medical care compared to decedents, and survivors’ medical costs did not differ across age categories. Further, since LTC increases with age for the surviving elderly, the total healthcare cost of survivors was found to be approximately half that of the decedents in the case of people aged 85 and over. Our findings regarding the pattern of medical expenditure divided by LTC use status differ from those reported by Werblow, *et al.* [19]. Using Swiss data, they found that LTC non-use was related to larger annual expenditure among decedents. Our results, however, show the opposite. The Swiss case revealed that LTC users, both decedents and survivors, incurred less medical expenditure compared to LTC non-users. Again, our results do not show such an effect of LTC use status. This difference between the current findings and those based on the Swiss data is not surprising, since LTC services in the Swiss data correspond to medical services for chronic care in Japan. The Swiss data did not include custodial and chronic care provided in homes and institutions, whereas the Japanese and The Netherlands data included both medical and LTC, and therefore provide a more comprehensive measure of the “total health cost” at the end of life.

If we consider only medical expenditures, then TTD and survivorship clearly affect the amount of expenditure, and age *per se* is negatively associated with expenditure, potentially supporting the “red herring” theory. However, our results, in accord with those from the Netherlands, suggest that although TTD is a strong predictor of higher medical expenditure among the elderly, and age *per se* is negatively correlated with medical expenditure among decedents, LTC that increases with age for decedents and survivors does contribute to a higher total healthcare cost among the elderly.

It could be argued, however, that an increase in the use of LTC with age might also be influenced by TTD, since the probability of death is higher for older people. Though this argument may have some validity, we cannot completely separate TTD from age in later life, because it is difficult to predict. In this study, the survivors had survived at least for 12 months. In spite of this, their total healthcare cost differed from that of their decedent counterparts in the same age category.

These findings have important implications for medical and LTC policy in Japan. The current results suggest that population aging results in increased demand and expenditure for LTC. Thus, substituting LTC for medical care at the end of life may not automatically lead to savings in resource use. Another implication of the current findings is that contribution of aging to rising healthcare expenditure should be distinguished according to survivorship. Total healthcare cost was found to be constant or to slightly decrease with age in decedents, suggesting that the cost until death is relatively stable among the elderly, while the risk of healthcare cost for survivors is proportional to age. This distinction should be taken into consideration in the design of insurance schemes, to appropriately cover healthcare costs among the elderly.

Some limitations of the current study should be taken into consideration. First, the data did not contain any relevant information regarding the quality of healthcare provided. Whether the quality of service in medical and LTC services differ across age and survivorship categories is an issue that requires further investigation. Moreover, the generalizability of our findings is limited because our data was derived from an insurer in a single prefecture. As such, larger-scale data collection from multiple insurers is necessary to confirm these results. In the statistical methodology we adopted, we could have used more sophisticated econometric estimation techniques to determine the probability and amount of service use, including regression models. We chose not to do so because our dataset contained only age and sex as potential regressor variables. More detailed data on the characteristics of beneficiaries, especially on health status and detailed diagnoses would be helpful to further clarify the pattern of healthcare resource use in later life.

## Figures and Tables

**Table 1.** Characteristics of decedent and survivor beneficiaries aged 65 and over.

	**Decedent**	**%**	**Survivor**	**%**	**p value (χ^2^ test)**

Sampled year	2001–2003		Apr.2002–Mar.2003		
Total number	50,857		364,484		

Age					
65–74	8,558	(16.8)	125,941	(34.6)	
75–84	19,968	(39.3)	177,720	(48.7)	
85–	22,331	(43.9)	60,823	(16.7)	<0.001

Gender					
female	25,726	(50.6)	218,638	(60.0)	<0.001

Recipients of care types					
outpatient	40,575	(79.8)	344,051	(94.4)	<0.001
inpatient	38,833	(76.4)	87,949	(21.1)	<0.001
home care	16,341	(32.1)	43,345	(11.9)	<0.001
institutional care	12,588	(24.8)	15,868	(4.4)	<0.001

Death place					
nursing homes	6,218	(12.2)			
hospitals	35,199	(69.2)			
other than nursing homes/hospitals (including at home)	9,440	(18.6)			

**Table 2.** Estimated average annual expenditure of decedent and survivor beneficiaries aged 65 and over.

**Age 65–74**	**Male**		**Female**	
	**Survivor**	**Decedent**	**Survivor**	**Decedent**
**outpatient**	439,493	626,356	403,907	566,967
**inpatient**	307,130	2,802,176	224,355	2,644,243
**homecare**	39,480	112,450	39,058	142,087
**institutional care**	29,631	166,297	33,022	256,836
**Age 75–84**				

**outpatient**	450,215	540,332	392,331	377,010
**inpatient**	336,159	2,408,123	297,274	2,061,216
**homecare**	53,913	173,560	94,900	212,864
**institutional care**	43,266	315,442	107,735	611,494
**Age 85–**				

**outpatient**	398,789	366,463	288,978	232,770
**inpatient**	500,118	1,847,853	536,086	1,542,349
**homecare**	151,674	301,256	224,418	287,824
**institutional care**	164,352	667,081	463,933	1,209,633

Unit; Japanese Yen (JPY; 100 JPY corresponds to approximately 1 USD)
